# Seroprevalence against *Rickettsia* and *Borrelia* Species in Patients with Uveitis: A Prospective Survey

**DOI:** 10.1155/2017/9247465

**Published:** 2017-11-26

**Authors:** Kim B. Madsen, Katarina Wallménius, Åke Fridman, Carl Påhlson, Kenneth Nilsson

**Affiliations:** ^1^Section of Opthalmology, Falu Hospital, Falun, Sweden; ^2^Department of Medical Sciences, Section of Clinical Microbiology, Uppsala University, Uppsala, Sweden; ^3^Department of Medical Sciences, Section of Infectious Diseases, Uppsala University, Uppsala, Sweden; ^4^Centre of Clinical Research, Falu Hospital, Falun, Sweden

## Abstract

Vector-borne diseases such as Lyme borreliosis and rickettsioses have been associated with ocular inflammation. Our aim was to study patients with diagnosed uveitis to evaluate serological signs of infection or exposure to these tick-borne agents. Forty-eight patients were prospectively examined with serology together with medical records and a questionnaire concerning previous exposure, diseases, and treatments. Seven patients (14.6%) showed seroconversion to *Rickettsia* spp. between acute and convalescent phase sera, which provides support for a positive *Rickettsia* diagnosis according to guidelines. The specificity was confirmed by Western blot. Additional 28 patients had stationary titres of which eight (16.6%) had 1 : 256 or higher titre in the first serum, and another 13 patients were seronegative. No epidemiological risk factor or marker could be identified. For *Borrelia*, only three patients showed moderate IgG titres. A control group of 100 blood donors, 60 patients with rheumatic disease, and 56 patients seeking medical care were tested of which 2.0–7.1% showed low anti-*Rickettsia* titres and 3.0–8.3% anti-*Borrelia* titres. The findings are indicative for an association between infection or exposure to *Rickettsia* spp. and uveitis with a seropositivity among patients with recurrent uveitis in concordance with the spread of rickettsial exposure in a tick-exposed population.

## 1. Introduction

The lifetime incidence of definite acute anterior uveitis (AAU), characterized by inflammation of the uvea, is approximately 0.2% in the general population and 1% in the histocompatibility antigen HLA-B27-positive population [1–3]. Classical signs of AAU are a red painful eye, photophobia, blurred vision, floaters, and sometimes loss of peripheral vision [4, 5]. Chronic uveitis, on the other hand, may be symptomless in the initial stages but can ultimately lead to permanent vision loss [4, 6–8].

The known aetiologies of uveitis fall into three main categories: (a) infectious, including viruses and bacteria, where the latter may be a contributing factor in combination with HLA-B27, (b) systemic inflammatory disease, and (c) malignant and leukemic cells, lymphoma, and melanoma [8–10]. However, the aetiology often remains unknown and the lack of specific pathognomonic signs of certain infectious agents is not uncommon [4, 11, 12].

Rickettsioses are systemic infections with symptoms caused by vasculitis due to the bacterial marked tropism for the endothelial cells of small vessels. The Gram-negative *Rickettsia* bacteria are arthropod vector-borne and worldwide distributed [13, 14]. Ocular manifestations such as scotoma, floaters, redness, or decreased vision are reported in *Rickettsia* infections but are usually self-limited and may easily be overlooked. Of the spotted fever *Rickettsia* (SFR), *Rickettsia conorii* and *R. rickettsii* have been reported as the cause of mainly posterior uveitis, with a chorioretinal involvement [15, 16].

In Sweden, *R. helvetica*, is, besides a single finding of *R. sibirica*, the only reported tick-transmitted SFR, occurring in approximately 5–10% of *Ixodes ricinus* ticks [17]. Previous serosurveys in Sweden have shown IgG antibodies to *Rickettsia* spp. in 3.0–44.0% of tick-exposed subjects, compared to 2–43.5% to *Borrelia* spp. [18–23]. Reported patients exposed to *Rickettsia* spp. have shown variable usually self-limiting unspecific symptoms such as flu-like fever, but sometimes more severe symptoms like meningitis or facial paralysis [24–26]. Moreover, the louse-borne *R. felis* has been detected in Sweden in patients with neurological symptoms, but thus far, *R. felis* has not been reported in any vector in Sweden [27].

The aim was to prospectively study patients presenting symptoms of and being diagnosed with uveitis, regardless of localization in the eye, to investigate if serological signs of *Borrelia* or *Rickettsia* infection or exposure are associated with acute or recurrent uveitis, for what would otherwise be considered a noninfectious uveitis.

## 2. Patients and Methods

### 2.1. Patients

A total of 48 patients diagnosed with uveitis, representing 1/3 of all patients with that diagnosis during 2013 and 2014, at the Ophthalmological Clinic, Falun Hospital, were after acceptance of informed consent included in the study, regardless of which part of the eye that was affected. Patients were sampled for two sera (S1-S2): sample 1 (S1) within the first 2 weeks of presentation and S2 up to 4–6 weeks later. Some patients who were treated had additional titres drawn (S3-S4). All patients that fulfilled the selection criteria, comprising that the uveitis had to be primary (acute, recurrent, or chronic) and not a secondary complication to intraocular surgery, corneal infections, etc., were included in the study. All patients were given a complete ocular examination at every visit, that is, visual acuity, slit lamp biomicroscopy examination (including indirect slit lamp biomicroscopy of the posterior pole), and tonometry. The uveitis was classified according to accepted practices (SUN working group definitions) as anterior, intermediate, posterior, or panuveitis.

All included patients were asked to answer a questionnaire regarding prior tick or flea bite, frequent visits to forest/rural areas, exposure to animals, past episodes of uveitis, rheumatic and autoimmune diseases, prior testing for HLA-B27, sarcoidosis, tuberculosis, inflammatory bowel disease, and antibiotic treatment. Information concerning previous and actual symptoms and diagnoses according to the questions in the questionnaire, laboratory findings, and initial treatment were obtained from the medical records which verified or expanded the information given.

Treatment primarily consisted of topical steroids (dexamethasone) and topical cycloplegic agents (cyclopentolate). Only a few patients required oral steroid treatment, and one patient was on a methotrexate regimen at the time of testing, an option for controlling uveitis recurrence [28].

Antibiotic treatment in the form of doxycycline (100 mg orally twice a day), for 14 days, was given to patients who had high initial titres (>1 : 256) against *Rickettsia* spp. or confirmed 4-fold rise in titre after the analysis of sample two (S2). Some of these patients were also followed with additional serum samples (S3, S4) for the determination of titre after completion of treatment. As a control group, from the same geographical area, sera from 100 healthy blood donors were randomly chosen together with samples from 60 patients diagnosed with rheumatic disease and 56 patients representing patients who had sought medical care for general medical reasons. The latter group represents patients with scheduled visits for blood pressure controls and renewal of prescriptions, namely, patients that did not show any sign of infection, and the blood samples were taken as part of the regular visit. Patients with rheumatic background were chosen as controls to examine their seroreactivity in general, although the reactivity might represent unspecific reactions, and if these patients differ from patients without rheumatic disease or ongoing infection.

### 2.2. Serology (Immunofluorescence Assay)

*R. helvetica*-infected Vero cells supplemented with 10% yolk sac solution were used as the bacterial antigen for an immunofluorescence assay (IFA), as previously described [18, 19]. According to the guidelines for the diagnosis of tick-borne bacterial diseases in Europe, IgG titers ≥ 1 : 128 and/or IgM titers ≥ 1 : 64 and/or a fourfold increase in two sera within a 2- to 4-week interval is considered indicative of infection by *Rickettsia* spp. if homologous antigens are used in the IFA test. The patients were divided into four groups based on their serologic results: group 1—a fourfold or greater rise in IgG titre between acute phase (S1) and convalescent phase (S2) sera was tested in parallel; group 2—single IgG endpoint titres of ≥1 : 256 in S1 or S2 were considered presumptive evidence of recent or current exposure; group 3—single IgG and/or IgM endpoint titres ≥ 1 : 64 and <1 : 256, respectively, were regarded as supportive evidence indicative of either past exposure or early response to exposure; and group 4—titres < 1 : 64 were considered negative for IgG/IgM [26]. Persisting IgM antibodies alone was interpreted as nonspecific cross-reactivity due to exposure to other organisms and autoimmune responses or possibly as a sign of a previous exposure. A fourfold increase in titre is, according to the guidelines, a criterion that provides a strong support for positive *Rickettsia* spp. diagnosis along with epidemiological, clinical, laboratory, and bacteriological findings [29]. A serum sample from a patient with proven endpoint IgG/IgM titres of 1 : 512/1 : 128, respectively, to *R. helvetica* was used as a positive control and as a negative control of human blood donor serum. When discrete structures that were morphologically compatible with *Rickettsia* were visible with ≥2^+^ brightness, the sample was considered to be positive. Laboratory evidence of current or previous infection with *B. burgdorferi* (IgG/IgM) was based on the analysis of serum samples using a commercial enzyme-linked immunosorbent assay (ELISA), according to the manufacturer's instructions (Euroimmun AG (Aktiengesellschaft), Lübeck, Germany). For the 48 patients included in the study, only serum two (S2) was tested for *Borrelia*.

### 2.3. Western Blot (WB)

Sera from seven of the IgG-positive patients (pat. numbers 1–7, group 1) were diluted to titres 1 : 200 and tested for WB with *R. helvetica* whole cell antigen using Amersham WB system (GE Healthcare) in accordance with the manufacture's instructions. Serum from a patient previously proven to have a rickettsial infection with high antibody titres in IFA (IgG 1 : 128) was used as the positive control. A serum from a healthy blood donor and the secondary antibody alone served as the negative controls.

### 2.4. Statistical Analyses

Standard parametric statistics (confidence interval according to Fleiss with Yates correction) were used for continuous variables, giving a mean + 95% confidence interval (CI). Fisher's exact test and chi-square test (*χ*^2^) were used to compare the proportions, and a *p* value < 0.05 was considered statistically significant. Statistical analyses were conducted using Predictive Analytics Software (PASW®) Statistics 20.

## 3. Results

### 3.1. Patients

The age distribution was between 13 and 77 years (mean age 53 years and median age 53.5 years) including 20 females and 28 males. Most patients had sought medical care within one week, usually one to two days after symptom onset. All sera were examined for the presence of rickettsial antibodies, and all patients were analysed for *Borrelia* spp. antibodies in serum S2. None of the patients underwent lumbar puncture. One of the included patients was excluded when it was discovered that the diagnosed uveitis was secondary to cataract surgery as a result of corneal injury. This patient was tested negative for rickettsial antibodies. Five other patients dropped out of the study; they changed their decision to participate or failed to leave samples during the defined time period. Of the patients in the rheumatic control group (median age 53, 19 men and 41 women), half was tested positive for rheumatoid factor and anticyclic citrullinated peptides (anti-CCP) and the other half showed the presence of antinuclear antibodies (ANA) or ENA, that is, antibodies to extractable nuclear antigens. None of the 56 patients (median age 56, 22 men and 34 women), representing patients who had sought medical care for general medical reasons showed any sign of infection and had normal values of C-reactive protein (<10 mg/L).

### 3.2. Serology

The laboratory results and details from the medical record of each patient are summarized in Tables 1 and 2. Of the 48 patients with uveitis, seven patients (14.6%) (numbers 1–7) showed a fourfold rise in IgG titre between S1 and S2 (Table 1). Of the remaining 41 patients, twelve patients (25%) (numbers 8–19) presented IgG/IgM titres ≥ 1 : 128 indicating a recent or current exposure, of which eight had IgG titres ≥ 1 : 256 in the first serum; sixteen patients (33.3%) (numbers 19–35) had threshold titres between ≥1 : 64 and ≤1 : 128 as a result supportive of early response (IgM), past exposure, or nonspecific reactivity (IgM); and 13 patients (27.1%) (numbers 35–48) were seronegative (<1 : 64). Of all the 48 patients analysed for convalescent sera (S2), three (numbers 1, 18, and 30) had IgG antibodies against *Borrelia* spp. in moderate titres and three patients (numbers 16, 20, and 28) showed a slight seroreactivity against IgM for *Borrelia* spp. as well. Among the blood donors, two of 100 were seropositive in IFA against *Rickettsia* spp. with antibody titres at most 1 : 64; three of the 60 (5%) patients with rheumatoid arthritis and four of the 56 (7.1%) patients seeking general medical care presented IgG antibodies of at most 1 : 64 except for one case where the titre was 1 : 128. The corresponding findings for *Borrelia* spp. in each group were three (3%), five (8.3%), and four patients (7.1%), showing only low to moderate antibody levels of IgG of which two individuals also had low IgM titres.

### 3.3. Western Blot

**Figure 1:**
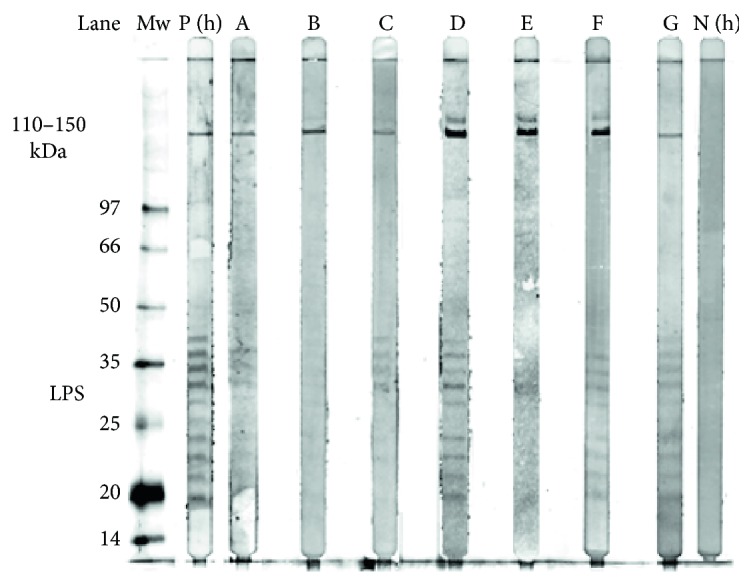
Western blot analysis of IgG antibodies against *R. helvetica* whole cell antigen. Lane A–G demonstrates the lipopolysaccharide ladders and specific reactions against *R. helvetica* proteins in the 110–150 kDa region for serum S2 and S3 (patient 3) for patients 1–7, in titres 1 : 200. Lane P(h) demonstrates specific proteins and the lipopolysaccharide (LPS) ladders reacting with a human antiserum from a patient diagnosed with rickettsial infection used as a positive control (P(h)). As a negative control, a serum from a healthy human blood donor (N(h)) was used. Mw = molecular marker.

WB for patient numbers 1–7 showed a specific response against lipopolysaccharide (LPS) and protein antigens in the 110–150 kDa region for IgG to whole cell antigen of *R. helvetica* (Figure 1). Negative controls in the form of serum from a healthy blood donor and IFA negative patient showed no specific reactions.

### 3.4. Medical Records and Questionnaire

Of the 48 patients in the study, 45 were diagnosed with uveitis engaging the anterior part of the eye, one with intermediate uveitis, and two patients primarily had a posterior involvement of the inflammation (Table 2). Thirteen patients were diagnosed as acute, 27 as recurrent acute, and eight as chronic uveitis in accordance with the criteria of the SUN Work Group [5].

Only patients with chronic uveitis had persistent inflammation after 3 months. In group 1 (pat. numbers 1–7), only one of seven patients (pat. number 5) had no prior history of uveitis but developed a chronic uveitis that still remained after three months. Two patients had previously been diagnosed with chronic uveitis, and the other four patients had an acute relapse of a recurrent uveitis. In groups 2–4 (pat. numbers 8–48), fifteen patients had no prior history of uveitis, and five patients were diagnosed with chronic uveitis of which one was a posterior and another an intermediate uveitis. Thirteen patients had acute first time iritis. The remaining 23 patients all had acute relapses of recurrent uveitis (Table 2).

The questionnaire and medical records showed that for 17 of the patients a probable cause of uveitis had previously been determined. These causes consisted of systemic inflammatory disease, IBD, sarcoidosis, Fuchs heterochromic iridocyclitis, and white dot syndrome. The remaining 31 causes were judged as idiopathic (Table 2). The outcome in groups 1–4 of the requested connections in the questionnaire is illustrated in Table 2. No significant statistical differences in the prevalence of symptoms between group 1 and group 4 could be demonstrated. Thirteen patients who previously stated being bitten by a tick were evenly distributed between the groups.

## 4. Discussion

The present study shows that of 48 patients with primary uveitis, 14.6% showed seroconversion with a fourfold increase in titre between two sera and another 16.6% initially high titres (≥1 : 256); together, 31.2% [CI 7.4–40.6] as serological supportive evidence of an underlying rickettsial infection or exposure might be associated to the ocular inflammation. In 7 patients (group 1), the specificity of the serological reaction was demonstrated by Western blot. According to the guidelines [29], the patients in group 1 fulfill criteria that give support to a positive *Rickettisa* spp. diagnosis. After the initiation of antibiotic treatment, the antibody titres fell one to several steps from the highest measured titre during the observation period, for five out of seven of these patients. In group 2, most of the patients presented an elevated titre (≥256) in S1, which in itself is a criterion for current infection but, in accordance with guidelines, other criteria need to be fulfilled to ensure a positive *Rickettsia* diagnosis. The antibody levels in group 2 remained elevated for many of the patients during the observation period, despite ongoing treatment. Nine out of twelve of these patients were HLA-B27 positive or had a rheumatic disease, two patients were not tested for HLA-B27 or rheumatic disease, and one was lacking signs of rheumatic disease. We therefore believe that the serological outcome in group 2 is difficult to assess and may be a result of unspecific reactivity due to polyclonal activation. The findings in group 3 are judged to represent residual reactivity from previous exposure, and those in group 4 were completely serologically negative.

Of the 19 serologically positive patients in groups 1 and 2, only three had no prior history of uveitis. The others had a previous history of uveitis but were asymptomatic or had an acute relapse at the study entry. None of these patients did present classic findings of rickettsial disease such as rash or eschar. A recent study however supports the assumption that infection with *R. helvetica* is often a subclinical disease with nonspecific symptoms likely leading to underestimation of human cases [20]. The majority of the patients did have anterior uveitis, rather than the posterior manifestations that have been reported previously in association with *R. conorii* infection, but the outcome may have been affected by the fact that only one third of the total number of patients with uveitis was included in the study [15, 16]. The seroprevalence among patients with uveitis may seem high but is in accordance with previously reported findings (3–44%) of anti-*Rickettsia* spp antibodies among tick-exposed or *Borrelia*-positive patients in Sweden. The corresponding seroprevalence to *Borrelia*, in Swedish populations, is between 2% and 26% up to 43.5% in a single study [18–23]. In southern European countries, the documented seroprevalence to *Rickettsia* spp. is between 4 and 37% from different areas up to 73.5% reported from an area in Spain [30–32]. As a comparison, the seroprevalence among healthy blood donors in Sweden is previously found between 0.6 and 3% which can be seen as a probable baseline [18–23]. Although most of the patients with uveitis had not noticed any tick bite, the results provide support that *Rickettsia* exposure is common in the uveitis group, and some of them did also develop serological evidence indicative of infection. The crucial issue is of course if and how a *Rickettsia* spp. exposition affects the development of an uveitis; possible options are through a causal relationship due to a primary infection, or as a trigger of an immunological response, or not at all. In order to answer these questions, further research is needed.

Hypothetically, an ongoing active or sublinical infection might be a contributing mechanism of uveitis or a postinfectious immune-mediated phenomenon in the patients with IgG-positive titres that do not decline with time. Those with HLA-B27^+^ uveitis could also have reactive inflammation after infection. It may also be possible that anterior uveitis is secondary to other molecular mimicry mechanisms than secondary to rickettsial infection. None of the patients with uveitis had laboratory or clinical signs of infection with Lyme borreliosis or other known viruses or bacteria that can cause uveitis. The most interesting cohort for further studies to evaluate a connection to rickettsial exposure seems to be those who presented with acute primary or acute recurrent ocular disease and had serological evidence of recent infection or exposure. Performing rickettsia serology in all patients with uveitis seems costly and unnecessary but of value for the study of selected cohorts in order to better understand the underlying causes. Early diagnosis and treatment might also affect the disease progression and recurrence rate in the short and long term. Methods for testing other cell markers of immune activity than antibody response, for example, interferon gamma production would also be desirable to distinguish immunity from ongoing infection. It is also possible that PCR on ocular fluid can be of value.

Intraocular inflammation has been reported as a main manifestation of *R. conorii* or *Rickettsia* spp. infection in a handful of cases [15]. Besides iritis, also choroiditis, retinitis, vasculitis, and vitreous manifestations have been reported associated with rickettsial infections [15, 16, 33]. However, the ocular involvement in rickettsial infections may be subclinical and self-limited and therefore overlooked [15, 16]. Several other arthropod vector-borne diseases have been found associated with uveitis and other ocular manifestations including West Nile virus, dengue fever, Rift Valley fever, and Chikungunya [33]. Concerning Lyme disease, ocular manifestations have been reported but are not considered being a common cause of uveitis [1, 34, 35]. In a previous retrospective study, anti-*Borrelia* antibodies were found in 10% of the patients, but none had Lyme borreliosis according to CDC criteria and showed probably a serological response that mainly represented a past infection [35, 36]. A similar outcome is shown in our study, in which 3 of the 48 patients showed low IgG titres indicative of previous exposure to Lyme disease and comparable to the findings in the control group representing the expected level of exposure.

About 1% of HLA-B27-positive patients develop AAU, and the risk is about ten times higher than that for an HLA-B27-negative person. HLA-B27-positive AAU is shown to be more severe, especially occurring in males, and probably has a somewhat better ophthalmological prognosis but is more strongly associated with ankylosing spondylitis or reactive arthritis [37]. The majority, 12 of 19, HLA-B27-positive patients presenting AAU in this study were male, and seven also had an associated rheumatic disease. The distribution of patients with a medical history regarding previous rheumatic disease or HLA-B27-positive and HLA-B27-negative patients was apparently dominant in group 2. No obvious risk factors or markers were identified after reviewing the questionnaires, and no significant difference in exposure was seen [15, 16, 37, 38]. It is well known in southern European countries that *Rickettsia* infections might give ocular manifestations [15, 39]. The corresponding situation in Sweden has not been studied before. However, the current study provides clear indications of a relationship between rickettsia exposure and uveitis [29]. The study is a pilot study, where several issues concerning prevalence, treatment, and general guidelines for treatment need to be answered through further studies. Among other things, the effect of antibiotics and its effect on the disease progression in the short and long term needs to be evaluated. Doxycycline 100 mg 1 × 2 is usually standard for treatment for 10–14 days, but longer treatment times of 8–10 weeks for ocular manifestations have been reported [33, 39, 40]. Because topical steroids were given as eye drops, it makes it more difficult to determine the antibiotic effect. An early treatment for rickettsial infections is generally of value, but the way in which antibiotics should be used for suspected rickettsial infection in ocular disease has to be further studied.

In conclusion, the current study reveals a possible association between uveitis and serological evidence of rickettsial exposure with a prevalence of seropositivity among patients with recurrent uveitis in concordance with the spread of rickettsial exposure in a tick-exposed population. However, further studies are required to understand the impact of these findings.

## Figures and Tables

**Table 1:** Results of serology for the *Rickettsia* spp. in serum S1–S5 and *Borrelia* spp. in S2 for all patients diagnosed with uveitis.

Group	Pat. number	Gender/age	S1		Weeks	S2		Weeks	S3		Weeks	S4		Weeks	S5		Borrelia/s (S2) IgG/IgM
			IgG	IgM	S1–S2	IgG	IgM	S1-S3	IgG	IgM	S1-S4	IgG	IgM	S1-S5	IgG	IgM	
1	1	M/64	64	<64	5	512	64	17	256	<64							Pos/border
	2	M/41	<64	64	14	128	256	45	64	128							Neg/neg
	3	F/13	<64	128	5	<64	512	10	256	512	26	512	1024	51	128	256	Neg/neg
	4	F/51	<64	512	4	128	128	17	128	<64							Neg/neg
	5	F/72	64	64	4	256	128	20	128	<64							Neg/neg
	6	M/69	<64	<64	8	256	<64	20	256	<64	32	128	<64				Neg/neg
	7	F/43	64	512	6	256	1024	22	256	256							Neg/neg

2	8	F/69	512	512	5	512	512	20	128	64							Neg/neg
	9	M/65	512	512	5	512	512	19	1024	2048	34	256	256				Neg/neg
	10	M/39	128	64	9	64	<64	16	256	512	33	64	128				Neg/neg
	11	F/66	1024	1024	5	1024	2048	19	512	512	33	256	256				Neg/neg
	12	M/66	512	256	4	1024	128	24	256	256							Neg/neg
	13	M/52	128	256	12	256	512	27	128	512	48	128	128				Border/neg
	14	F/60	256	512	5	256	128	22	256	128	32	256	128				Neg/neg
	15	M/58	128	<64	5	256	<64	21	<64	<64							Neg/neg
	16	M/50	256	<64	11	64	<64										Neg/pos
	17	M/37	256	256	6	128	128	18	128	128							Neg/neg
	18	F/59	128	<64	7	256	<64	25	128	<64							Pos/neg
	19	F/38	256	256	11	512	256										Neg/neg

3	20	F/77	64	<64	5	64	<64										Neg/pos
	21	M/55	64	<64	6	64	<64										Neg/neg
	22	M/48	64	<64	6	128	<64	19	64	<64							Neg/neg
	23	F/68	<64	128	5	<64	<64										Neg/neg
	24	M/71	128	128	4	128	64	23	64	<64							Neg/neg
	25	M/26	64	<64	12	64	<64										Neg/neg
	26	F/52	128	512	12	128	64	34	64	<64							Neg/neg
	27	M/38	64	<64	5	128	64	21	<64	<64							Neg/neg
	28	F/63	64	<64	6	64	<64										Neg/pos
	29	F/44	64	64	5	<64	64										Border/neg
	30	M/67	128	<64	4	64	<64										Pos/neg
	31	F/25	64	<64	7	64	<64										Neg/neg
	32	M/42	64	<64	7	64	<64										Neg/neg
	33	M/62	128	64	17	64	<64										Neg/neg
	34	F/18	<64	<64	5	64	64										Neg/neg
	35	F/54	128	128	9	64	128	14	128	128							Neg/neg

4	36	M/72	<64	<64	9	<64	<64										Neg/neg
	37	F/65	<64	<64	10	<64	<64										Neg/neg
	38	M/27	<64	<64	5	<64	<64										Neg/neg
	39	M/47	<64	<64	5	<64	<64										Neg/neg
	40	M/70	<64	<64	4	<64	<64										Neg/neg
	41	F/58	<64	<64	6	<64	<64										Neg/neg
	42	F/53	<64	<64	6	<64	<64										Neg/neg
	43	M/64	<64	<64	10	<64	<64										Neg/neg
	44	M/53	<64	<64	27	<64	<64										Neg/neg
	45	M/60	<64	<64	4	<64	<64										Neg/neg
	46	M/53	<64	<64	5	<64	<64										Neg/neg
	47	M/33	<64	<64	6	<64	<64										Neg/neg
	48	M/47	<64	<64	6	<64	<64										Neg/neg

Pat. number: patient number; M: male; F: female; S1–S5: serum samples 1–5.

**Table 2:** Summation of the outcome from medical records and questionnaires in groups 1–4.

Group	Pat. number	Inflamed part of the eye	Previous uveitis	Possible cause uveitis	Acute/chronic	Contact with fur animals	Often staying in the forest	Known tick bite	Known flea bite	IBD	Rheumatic disease	TB	Sarcoidosis	HLA-B27^+^	Antibiotic treatment last month
1	1	ANT	Y	ID	A	Ca	Y	Y	N	N	N	N	ND	Y	N
	2	ANT	Y	ID	A	N	N	N	N	N	ND	N	ND	Y	N
	3	ANT	Y	RD	C	N	N	Y	N	N	Y	N	ND	ND	N
	4	ANT	Y	ID	C	Ca	Y	N	Y	N	N	N	ND	ND	T1
	5	ANT	N	FU	C	O	Y	N	N	N	N	N	N	N	T2
	6	ANT	Y	MB	A	Ca + Do	Y	N	N	N	Y	N	ND	ND	N
	7	ANT	Y	ID	A	Ca	Y	Y	N	N	N	N	ND	N	N

2	8	ANT	N	IBD	A	N	Y	N	Y	Y	N	N	ND	ND	N
	9	ANT	Y	MB	A	N	N	N	N	N	Y	N	ND	ND	N
	10	ANT	Y	ID	A	Ca	Y	N	N	N	N	N	ND	Y	N
	11	ANT	Y	ID	A	Ca + Do	Y	N	N	N	N	N	N	Y	N
	12	ANT	Y	MB	A	N	N	N	N	N	Y	N	ND	Y	N
	13	ANT	Y	RD	A	N	Y	N	N	N	Y	N	ND	ND	N
	14	ANT	Y	ID	A	Ca + Do	Y	Y	Y	N	N	N	ND	Y	N
	15	ANT	N	ID	A	N	Y	Y	N	N	N	N	ND	Y	N
	16	ANT	Y	ID	A	N	Y	Y	N	N	N	N	ND	Y	N
	17	ANT	Y	MB	A	N	N	Y	Y	N	Y	N	ND	Y	N
	18	ANT	Y	ID	A	N	N	N	N	N	N	N	ND	ND	N
	19	ANT	Y	ID	C	N	N	N	N	N	N	N	N	N	N

3	20	ANT	N	ID	A	N	N	N	N	N	N	N	ND	ND	N
	21	ANT	Y	ID	A	N	Y	N	N	N	N	N	ND	N	N
	22	ANT	N	ID	A	N	Y	N	N	N	N	N	ND	ND	N
	23	ANT	Y	RD	A	N	Y	Y	N	N	Y	N	N	Y	N
	24	ANT	Y	ID	A	N	Y	N	N	N	N	N	N	Y	N
	25	ANT	N	S	A	N	N	N	N	N	N	N	Y	N	N
	26	ANT	N	ID	A	N	N	N	N	N	N	N	ND	ND	N
	27	ANT	N	ID	A	N	N	N	N	N	N	N	ND	N	N
	27	ANT	Y	ID	A	N	Y	N	N	N	N	N	ND	N	N
	29	ANT	Y	ID	A	N	Y	N	N	N	N	N	ND	Y	N
	30	ANT	N	ID	A	N	Y	Y	N	N	N	N	ND	Y	N
	31	ANT	N	ID	A	N	Y	Y	N	N	N	N	ND	ND	N
	32	ANT	N	ID	A	Ca	N	Y	Y	N	N	N	ND	ND	N
	33	ANT	N	MB	A	N	Y	N	N	N	Y	N	ND	ND	N
	34	INT	N	ID	C	O	Y	Y	N	N	N	N	ND	ND	N
	35	ANT	Y	ID	A	N	N	N	N	N	N	N	N	Y	T3

4	36	ANT	Y	ID	C	N	N	N	N	N	N	N	ND	N	N
	37	POST	Y	ID	A	D	Y	N	N	N	N	N	N	N	N
	38	ANT	Y	ID	A	D	N	N	N	N	N	N	ND	Y	N
	39	POST	N	IBD	C	Ca	N	N	N	Y	N	N	N	N	N
	40	ANT	N	ID	A	N	Y	N	N	N	N	N	ND	ND	N
	41	ANT	Y	RD	A	N	Y	N	Y	N	Y	N	ND	Y	N
	42	ANT	Y	RD	A	Ca	Y	N	N	N	Y	N	ND	Y	N
	43	ANT	Y	RD	A	Ca	N	N	N	N	Y	N	ND	Y	N
	44	ANT	Y	ID	A	N	Y	Y	Y	N	N	N	ND	ND	N
	45	ANT	Y	ID	A	O	N	N	N	N	N	N	ND	Y	N
	46	ANT	Y	RD	A	Do	Y	Y	N	N	Y	N	ND	N	N
	47	ANT	N	ID	A	N	N	N	N	N	N	N	ND	ND	N
	48	ANT	Y	FU	C	Do + O	Y	N	N	N	N	N	ND	ND	N

ANT: anterior; POST: posterior; INT: intermediate; Y: yes; N: no; ID: idiopathic disease; RD: rheumatic disease; FU: Fuchs heterochromic iridocyclitis; IBD: inflammatory bowel disease; MB: Mb Bechterew; S: sarcoidosis; A: acute; C: chronic; Ca: cat; Do: dog; O: other ND: no data; T1: treatment 1 (fenoxymetylpenicillin); T2: treatment 2 (amoxicillin, bensylpenicillin, cefotaxim, erytromtcin, moxifloxacin); T3: treatment 3 (mecillinam, nitrofurantoin).
